# The genome sequence of the White-backed Marble, *Hedya salicella* (Linnaeus, 1758)

**DOI:** 10.12688/wellcomeopenres.19436.1

**Published:** 2023-05-15

**Authors:** Douglas Boyes, Zoe Goate, University of Oxford and Wytham Woods Genome Acquisition Lab, Darwin Tree of Life Barcoding collective, Wellcome Sanger Institute Tree of Life programme, Wellcome Sanger Institute Scientific Operations: DNA Pipelines collective, Tree of Life Core Informatics collective, Darwin Tree of Life Consortium

**Affiliations:** 1 UK Centre for Ecology & Hydrology, Wallingford, England, UK; 2 Wellcome Sanger Institute, Hinxton, England, UK

**Keywords:** Hedya salicella, White-backed Marble, genome sequence, chromosomal, Lepidoptera

## Abstract

We present a genome assembly from an individual male *Hedya salicella* (the White-backed Marble; Arthropoda; Insecta; Lepidoptera; Tortricidae). The genome sequence is 742.3 megabases in span. Most of the assembly is scaffolded into 25 chromosomal pseudomolecules, including the Z sex chromosome. The mitochondrial genome has also been assembled and is 16.3 kilobases in length. Gene annotation of this assembly on Ensembl identified 11,961 protein coding genes.

## Species taxonomy

Eukaryota; Metazoa; Ecdysozoa; Arthropoda; Hexapoda; Insecta; Pterygota; Neoptera; Endopterygota; Lepidoptera; Glossata; Ditrysia; Tortricoidea; Tortricidae; Olethreutinae; Olethreutini; *Hedya*; *Hedya salicella* (Linnaeus, 1758) (NCBI:txid1869985).

## Background

The White-backed Marble, *Heyda salicella* (Linnaeus, 1758) is a single brooded, common species of micro moth widely distributed across Europe and introduced in North America ( Gilligan *et al.*, 2020). This large and distinctive *Heyda* species is predominantly white with a mottled chestnut and grey thorax. It has a wingspan of 19–24 mm and has been recorded in flight from the months June through to September. *H. salicella* inhabits areas where food plants are abundant, with sightings recorded in marshy areas amongst willows, banks of streams, open woodland and occasionally parks and gardens. Larvae feed on spun shoots and folded leaves of *Salix* (willow, sallow) and *Populus* (poplar, aspen) species ( Kimber, 2023).

The genome of *H. salicella* was sequenced as part of the Darwin Tree of Life Project, a collaborative effort to sequence all named eukaryotic species in the Atlantic Archipelago of Britain and Ireland. Here we present a complete chromosome-level genome sequence for *H. salicella*, based on one male specimen from Wytham Woods, Oxfordshire, UK. This high-quality complete genome assembly of *H. salicella*, among a phylogenetically diverse set of insect orders, will yield genomes from closely related species, permitting valuable insights into genomic change over shorter time frames ( Mulhair & Holland, 2022), while resolving the biogeographic origin of morphologically similar populations in Europe and North America.

## Genome sequence report

**Figure 1.**
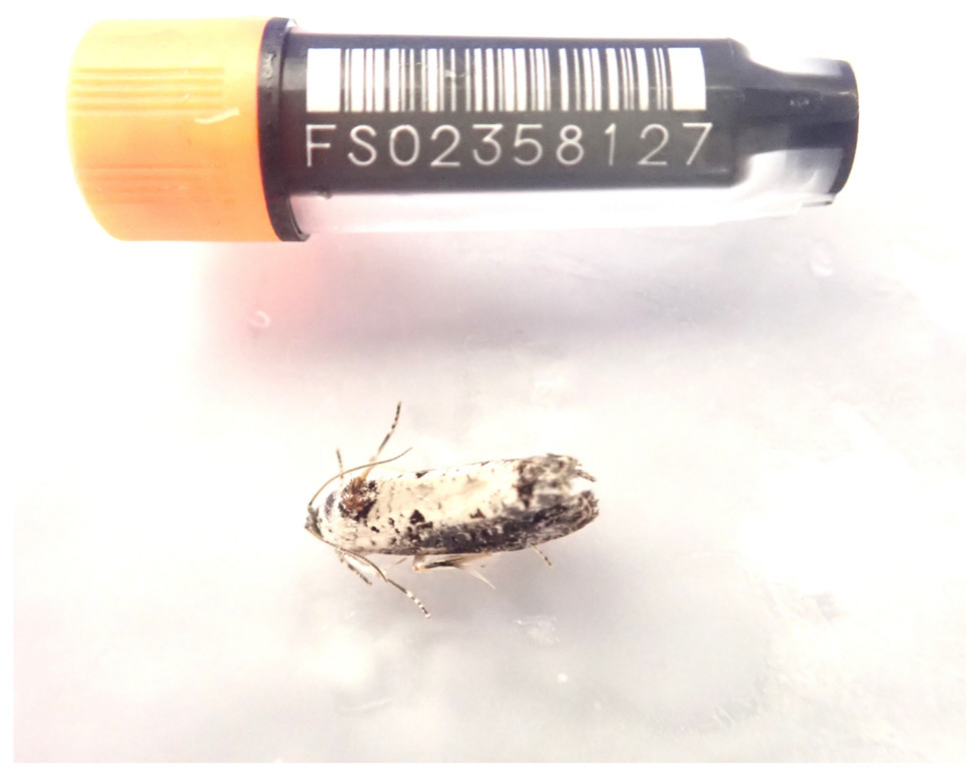
Photograph of the *Hedya salicella* (ilHedSali1) specimen used for genome sequencing.

The genome was sequenced from one male *Hedya salicella* ( Figure 1) collected from Wytham Woods, Oxfordshire, UK (latitude 51.77, longitude –1.34). A total of 25-fold coverage in Pacific Biosciences single-molecule HiFi long reads and 43-fold coverage in 10X Genomics read clouds were generated. Primary assembly contigs were scaffolded with chromosome conformation Hi-C data. Manual assembly curation corrected four missing joins or mis-joins and removed one haplotypic duplication, reducing the scaffold number by 16.67%.

**Figure 2.**
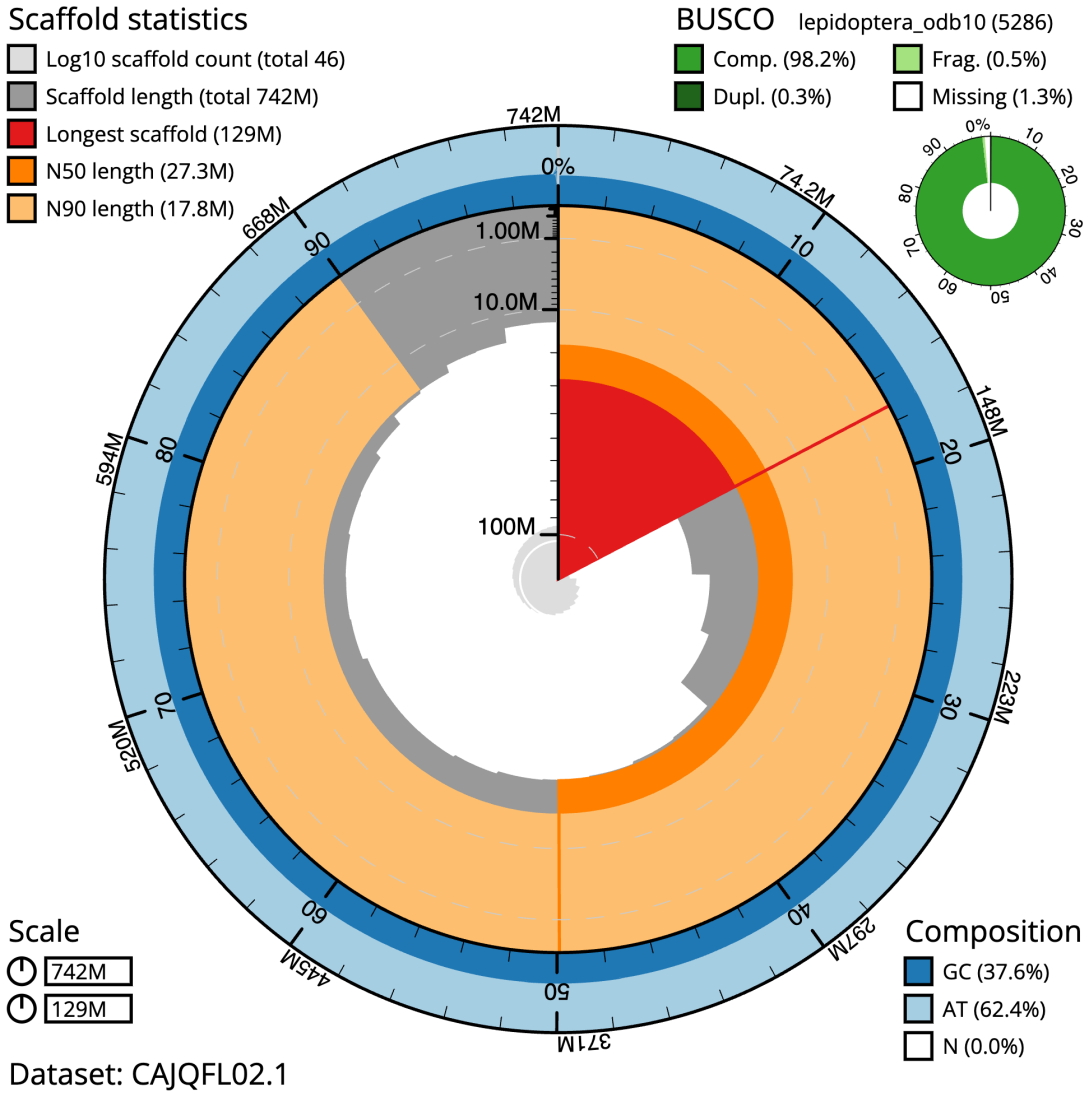
Genome assembly of *Hedya salicella*, ilHedSali1.2: metrics. The BlobToolKit Snailplot shows N50 metrics and BUSCO gene completeness. The main plot is divided into 1,000 size-ordered bins around the circumference with each bin representing 0.1% of the 742,325,546 bp assembly. The distribution of scaffold lengths is shown in dark grey with the plot radius scaled to the longest scaffold present in the assembly (128,845,201 bp, shown in red). Orange and pale-orange arcs show the N50 and N90 scaffold lengths (27,275,373 and 17,835,027 bp), respectively. The pale grey spiral shows the cumulative scaffold count on a log scale with white scale lines showing successive orders of magnitude. The blue and pale-blue area around the outside of the plot shows the distribution of GC, AT and N percentages in the same bins as the inner plot. A summary of complete, fragmented, duplicated and missing BUSCO genes in the lepidoptera_odb10 set is shown in the top right. An interactive version of this figure is available at https://blobtoolkit.genomehubs.org/view/ilHedSali1.2/dataset/CAJQFL02.1/snail.

The final assembly has a total length of 742.3 Mb in 45 sequence scaffolds with a scaffold N50 of 27.3 Mb ( Table 1). Most (99.88%) of the assembly sequence was assigned to 25 chromosomal-level scaffolds, representing 24 autosomes and the Z sex chromosome. Chromosome-scale scaffolds confirmed by the Hi-C data are named in order of size ( Figure 2– Figure 5; Table 2). While not fully phased, the assembly deposited is of one haplotype. Contigs corresponding to the second haplotype have also been deposited. The mitochondrial genome was also assembled and can be found as a contig within the multifasta file of the genome submission.

**Table 1.** Genome data for *Hedya salicella*, ilHedSali1.2.

Project accession data		
Assembly identifier	ilHedSali1.2	
Species	*Hedya salicella*	
Specimen	ilHedSali1	
NCBI taxonomy ID	1869985	
BioProject	PRJEB43799	
BioSample ID	SAMEA7520688	
Isolate information	ilHedSali1, male (whole organism)	
Assembly metrics *		*Benchmark*
Consensus quality (QV)	56	*≥ 50*
*k*-mer completeness	99.99%	*≥ 95%*
BUSCO **	C:98.2%[S:97.9%,D:0.3%], F:0.5%,M:1.3%,n:5,286	*C ≥ 95%*
Percentage of assembly mapped to chromosomes	99.88%	*≥ 95%*
Sex chromosomes	Z chromosome	*localised homologous pairs*
Organelles	Mitochondrial genome assembled	*complete single alleles*
Raw data accessions		
PacificBiosciences SEQUEL II	ERR6436368	
10X Genomics Illumina	ERR6054622–ERR6054625	
Hi-C Illumina	ERR6054619, ERR6054620, ERR6054621	
Genome assembly		
Assembly accession	GCA_905404275.2	
*Accession of alternate haplotype*	GCA_905404235.2	
Span (Mb)	742.3	
Number of contigs	60	
Contig N50 length (Mb)	25.6	
Number of scaffolds	45	
Scaffold N50 length (Mb)	27.3	
Longest scaffold (Mb)	128.9	
Genome annotation		
Number of protein-coding genes	11,961	
Number of non-coding genes	1,706	
Number of gene transcripts	20,143	

* Assembly metric benchmarks are adapted from column VGP-2020 of “Table 1: Proposed standards and metrics for defining genome assembly quality” from ( Rhie *et al.*, 2021). ** BUSCO scores based on the lepidoptera_odb10 BUSCO set using v5.3.2. C = complete [S = single copy, D = duplicated], F = fragmented, M = missing, n = number of orthologues in comparison. A full set of BUSCO scores is available at https://blobtoolkit.genomehubs.org/view/ilHedSali1.2/dataset/CAJQFL02.1/busco.

**Figure 3.**
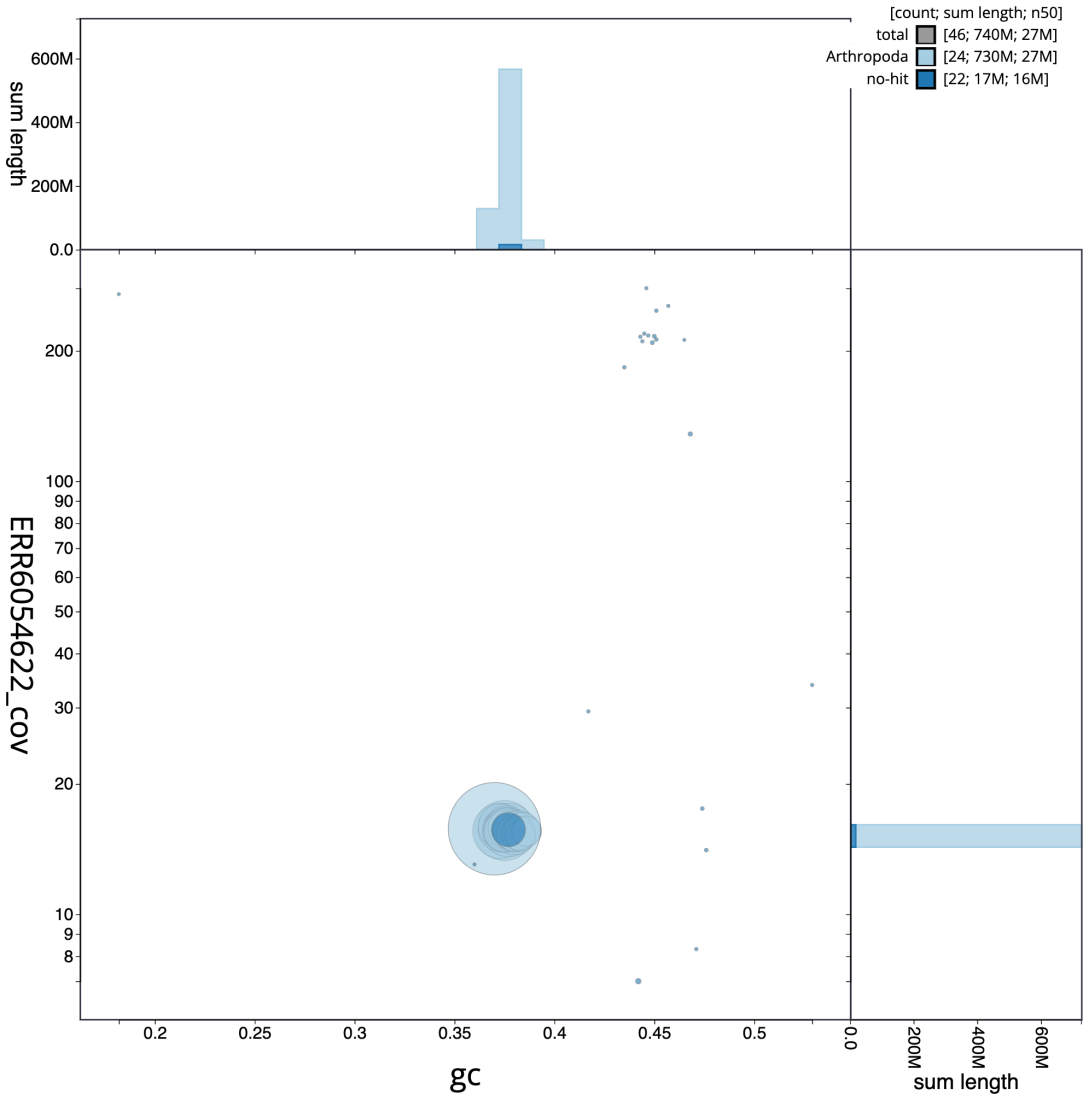
Genome assembly of *Hedya salicella*, ilHedSali1.2: BlobToolKit GC-coverage plot. Scaffolds are coloured by phylum. Circles are sized in proportion to scaffold length. Histograms show the distribution of scaffold length sum along each axis. An interactive version of this figure is available at https://blobtoolkit.genomehubs.org/view/ilHedSali1.2/dataset/CAJQFL02.1/blob.

**Figure 4.**
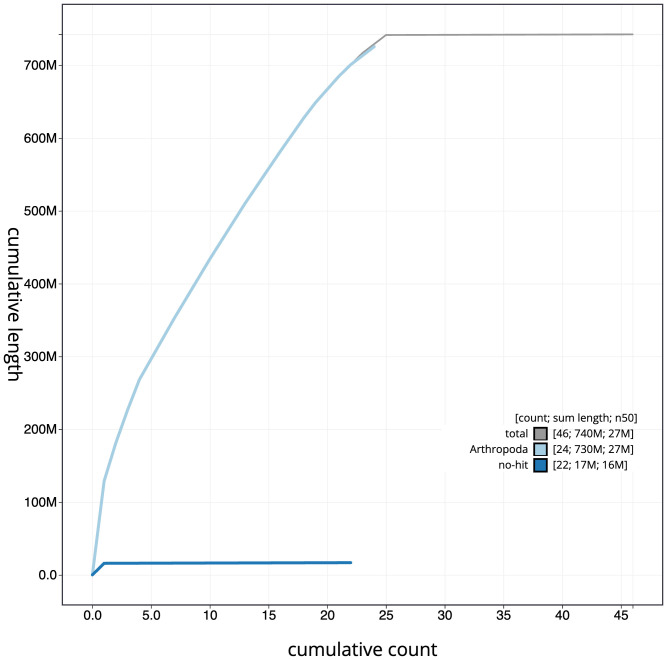
Genome assembly of *Hedya salicella*, ilHedSali1.2: BlobToolKit cumulative sequence plot. The grey line shows cumulative length for all scaffolds. Coloured lines show cumulative lengths of scaffolds assigned to each phylum using the buscogenes taxrule. An interactive version of this figure is available at https://blobtoolkit.genomehubs.org/view/ilHedSali1.2/dataset/CAJQFL02.1/cumulative.

**Figure 5.**
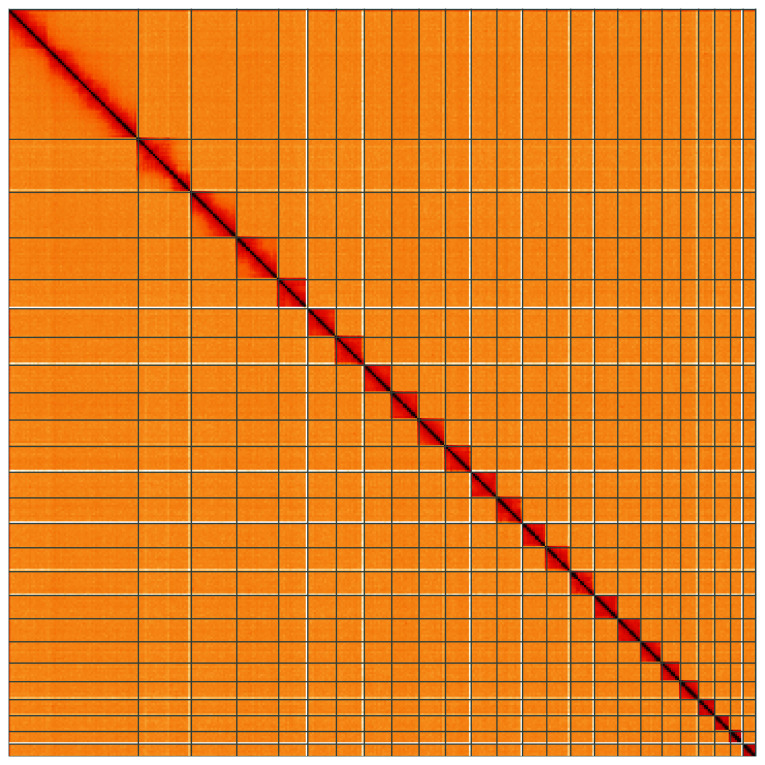
Genome assembly of *Hedya salicella*, ilHedSali1.2: Hi-C contact map of the ilHedSali1.2 assembly, visualised using HiGlass. Chromosomes are shown in order of size from left to right and top to bottom. An interactive version of this figure may be viewed at https://genome-note-higlass.tol.sanger.ac.uk/l/?d=dVzSopIPQm2BTrcTNwkrZw.

**Table 2.** Chromosomal pseudomolecules in the genome assembly of *Hedya salicella*, ilHedSali1.

INSDC accession	Chromosome	Size (Mb)	GC%
FR990097.1	1	52.61	37.5
FR990098.1	2	45.12	37.3
FR990099.1	3	41.55	37.7
FR990100.1	4	28.87	37.3
FR990101.1	5	28.41	37.3
FR990102.1	6	27.72	37.5
FR990103.1	7	27.28	38
FR990104.1	8	27.07	37.6
FR990105.1	9	26.26	37.5
FR990106.1	10	25.55	37.7
FR990107.1	11	25.49	37.6
FR990108.1	12	25.43	37.6
FR990109.1	13	24.05	37.5
FR990110.1	14	23.77	37.7
FR990111.1	15	23.63	38
FR990112.1	16	23.06	37.9
FR990113.1	17	22.87	38.2
FR990114.1	18	21.1	37.9
FR990115.1	19	18.48	38
FR990116.1	20	17.84	38.4
FR990117.1	21	15.95	37.7
FR990118.1	22	15.76	38.2
FR990119.1	23	12.43	38.3
FR990120.1	24	12.34	38.6
FR990096.1	Z	128.85	37
FR990121.1	MT	0.02	18.2
-	unplaced	0.84	45.2

The estimated Quality Value (QV) of the final assembly is 56 with *k*-mer completeness of 99.99%, and the assembly has a BUSCO v5.3.2 completeness of 98.2% (single = 97.9%, duplicated = 0.3%), using the lepidoptera_odb10 reference set ( *n* = 5,286).

Metadata for specimens, spectral estimates, sequencing runs, contaminants and pre-curation assembly statistics can be found at https://links.tol.sanger.ac.uk/species/1869985.

## Genome annotation report

The ilHedSali1.2, GCA_905404275.2 genome assembly was annotated using the Ensembl rapid annotation pipeline ( Table 1; https://rapid.ensembl.org/Hedya_salicella_GCA_905404275.2/Info/Index). The resulting annotation includes 20,143 transcribed mRNAs from 11,961 protein-coding and 1,706 non-coding genes.

## Methods

### Sample acquisition and nucleic acid extraction

A male *Hedya salicella* (specimen no. Ox000472, individual ilHedSali1) was collected from Wytham Woods, Oxfordshire (biological vice-county: Berkshire), UK (latitude 51.77, longitude –1.34) on 13 June 2020. The specimen was taken from woodland by Douglas Boyes (University of Oxford) using a light trap. The specimen was identified by the collector, and preserved on dry ice.

DNA was extracted at the Tree of Life laboratory, Wellcome Sanger Institute (WSI). The ilHedSali1 sample was weighed and dissected on dry ice with tissue set aside for Hi-C sequencing. Whole organism tissue was disrupted using a Nippi Powermasher fitted with a BioMasher pestle. High molecular weight (HMW) DNA was extracted using the Qiagen MagAttract HMW DNA extraction kit. Low molecular weight DNA was removed from a 20 ng aliquot of extracted DNA using the 0.8X AMpure XP purification kit prior to 10X Chromium sequencing; a minimum of 50 ng DNA was submitted for 10X sequencing. HMW DNA was sheared into an average fragment size of 12–20 kb in a Megaruptor 3 system with speed setting 30. Sheared DNA was purified by solid-phase reversible immobilisation using AMPure PB beads with a 1.8X ratio of beads to sample to remove the shorter fragments and concentrate the DNA sample. The concentration of the sheared and purified DNA was assessed using a Nanodrop spectrophotometer and Qubit Fluorometer and Qubit dsDNA High Sensitivity Assay kit. Fragment size distribution was evaluated by running the sample on the FemtoPulse system.

### Sequencing

Pacific Biosciences HiFi circular consensus and 10X Genomics read cloud DNA sequencing libraries were constructed according to the manufacturers’ instructions. DNA sequencing was performed by the Scientific Operations core at the WSI on Pacific Biosciences SEQUEL II (HiFi) and HiSeq X Ten (10X) instruments. Hi-C data were also generated from tissue of ilHedSali1 using the Arima2 kit and sequenced on the HiSeq X Ten instrument.

### Genome assembly, curation and evaluation

Assembly was carried out with Hifiasm ( Cheng *et al.*, 2021) and haplotypic duplication was identified and removed with purge_dups ( Guan *et al.*, 2020). One round of polishing was performed by aligning 10X Genomics read data to the assembly with Long Ranger ALIGN, calling variants with FreeBayes ( Garrison & Marth, 2012). The assembly was then scaffolded with Hi-C data ( Rao *et al.*, 2014) using SALSA2 ( Ghurye *et al.*, 2019). The assembly was checked for contamination and corrected using the gEVAL system ( Chow *et al.*, 2016) as described previously ( Howe *et al.*, 2021). Manual curation was performed using gEVAL, HiGlass ( Kerpedjiev *et al.*, 2018) and Pretext ( Harry, 2022). The mitochondrial genome was assembled using MitoHiFi ( Uliano-Silva *et al.*, 2022), which runs MitoFinder ( Allio *et al.*, 2020) or MITOS ( Bernt *et al.*, 2013) and uses these annotations to select the final mitochondrial contig and to ensure the general quality of the sequence.

A Hi-C map for the final assembly was produced using bwa-mem2 ( Vasimuddin *et al.*, 2019) in the Cooler file format ( Abdennur & Mirny, 2020). To assess the assembly metrics, the *k*-mer completeness and QV consensus quality values were calculated in Merqury ( Rhie *et al.*, 2020). This work was done using Nextflow ( Di Tommaso *et al.*, 2017) DSL2 pipelines “sanger-tol/readmapping” ( Surana *et al.*, 2023a) and “sanger-tol/genomenote” ( Surana *et al.*, 2023b). The genome was analysed within the BlobToolKit environment ( Challis *et al.*, 2020) and BUSCO scores ( Manni *et al.*, 2021; Simão *et al.*, 2015) were calculated.

Table 3 contains a list of relevant software tool versions and sources.

**Table 3.** Software tools: versions and sources.

Software tool	Version	Source
BlobToolKit	4.0.7	https://github.com/blobtoolkit/blobtoolkit
BUSCO	5.3.2	https://gitlab.com/ezlab/busco
FreeBayes	1.3.1-17-gaa2ace8	https://github.com/freebayes/freebayes
gEVAL	N/A	https://geval.org.uk/
Hifiasm	0.12	https://github.com/chhylp123/hifiasm
HiGlass	1.11.6	https://github.com/higlass/higlass
Long Ranger ALIGN	2.2.2	https://support.10xgenomics.com/genome-exome/ software/pipelines/latest/advanced/other-pipelines
Merqury	MerquryFK	https://github.com/thegenemyers/MERQURY.FK
MitoHiFi	2	https://github.com/marcelauliano/MitoHiFi
PretextView	0.2	https://github.com/wtsi-hpag/PretextView
purge_dups	1.2.3	https://github.com/dfguan/purge_dups
SALSA	2.2	https://github.com/salsa-rs/salsa
sanger-tol/genomenote	v1.0	https://github.com/sanger-tol/genomenote
sanger-tol/readmapping	1.1.0	https://github.com/sanger-tol/readmapping/tree/1.1.0

### Genome annotation

The Ensembl gene annotation system ( Aken *et al.*, 2016) was used to generate annotation for the *Hedya salicella* assembly (ilHedSali1.2, GCA_905404275.2). Annotation was created primarily through alignment of transcriptomic data to the genome, with gap filling via protein-to-genome alignments of a select set of proteins from UniProt ( UniProt Consortium, 2019).

### Ethics and compliance issues

The materials that have contributed to this genome note have been supplied by a Darwin Tree of Life Partner. The submission of materials by a Darwin Tree of Life Partner is subject to the Darwin Tree of Life Project Sampling Code of Practice. By agreeing with and signing up to the Sampling Code of Practice, the Darwin Tree of Life Partner agrees they will meet the legal and ethical requirements and standards set out within this document in respect of all samples acquired for, and supplied to, the Darwin Tree of Life Project. Each transfer of samples is further undertaken according to a Research Collaboration Agreement or Material Transfer Agreement entered into by the Darwin Tree of Life Partner, Genome Research Limited (operating as the Wellcome Sanger Institute), and in some circumstances other Darwin Tree of Life collaborators.

## Data Availability

European Nucleotide Archive: *Hedya salicella* (white-backed marble). Accession number PRJEB43799; https://identifiers.org/ena.embl/PRJEB43799 ( Wellcome Sanger Institute, 2022). The genome sequence is released openly for reuse. The *Hedya salicella* genome sequencing initiative is part of the Darwin Tree of Life (DToL) project. All raw sequence data and the assembly have been deposited in INSDC databases. Raw data and assembly accession identifiers are reported in Table 1.

## Author information

Members of the University of Oxford and Wytham Woods Genome Acquisition Lab are listed here: https://doi.org/10.5281/zenodo.4789928.

Members of the Darwin Tree of Life Barcoding collective are listed here: https://doi.org/10.5281/zenodo.4893703.

Members of the Wellcome Sanger Institute Tree of Life programme are listed here: https://doi.org/10.5281/zenodo.4783585.

Members of Wellcome Sanger Institute Scientific Operations: DNA Pipelines collective are listed here: https://doi.org/10.5281/zenodo.4790455.

Members of the Tree of Life Core Informatics collective are listed here: https://doi.org/10.5281/zenodo.5013541.

Members of the Darwin Tree of Life Consortium are listed here: https://doi.org/10.5281/zenodo.4783558.

## References

[ref-1] Abdennur N Mirny LA : Cooler: Scalable storage for Hi-C data and other genomically labeled arrays. *Bioinformatics.* 2020;36(1):311–316. 10.1093/bioinformatics/btz540 31290943 PMC8205516

[ref-2] Aken BL Ayling S Barrell D : The Ensembl gene annotation system. *Database (Oxford).* 2016;2016:baw093. 10.1093/database/baw093 27337980 PMC4919035

[ref-3] Allio R Schomaker-Bastos A Romiguier J : MitoFinder: Efficient automated large‐scale extraction of mitogenomic data in target enrichment phylogenomics. *Mol Ecol Resour.* 2020;20(4):892–905. 10.1111/1755-0998.13160 32243090 PMC7497042

[ref-4] Bernt M Donath A Jühling F : MITOS: Improved *de novo* metazoan mitochondrial genome annotation. *Mol Phylogenet Evol.* 2013;69(2):313–9. 10.1016/j.ympev.2012.08.023 22982435

[ref-5] Challis R Richards E Rajan J : BlobToolKit - interactive quality assessment of genome assemblies. *G3 (Bethesda).* 2020;10(4):1361–1374. 10.1534/g3.119.400908 32071071 PMC7144090

[ref-6] Cheng H Concepcion GT Feng X : Haplotype-resolved *de novo* assembly using phased assembly graphs with hifiasm. *Nat Methods.* 2021;18(2):170–175. 10.1038/s41592-020-01056-5 33526886 PMC7961889

[ref-7] Chow W Brugger K Caccamo M : gEVAL — a web-based browser for evaluating genome assemblies. *Bioinformatics.* 2016;32(16):2508–10. 10.1093/bioinformatics/btw159 27153597 PMC4978925

[ref-24] Di Tommaso P Chatzou M Floden EW : Nextflow enables reproducible computational workflows. *Nat Biotechnol.* 2017;35(4):316–319. 10.1038/nbt.3820 28398311

[ref-8] Garrison E Marth G : Haplotype-based variant detection from short-read sequencing. 2012. 10.48550/arXiv.1207.3907

[ref-9] Ghurye J Rhie A Walenz BP : Integrating Hi-C links with assembly graphs for chromosome-scale assembly. *PLoS Comput Biol.* 2019;15(8):e1007273. 10.1371/journal.pcbi.1007273 31433799 PMC6719893

[ref-10] Gilligan TM Brown JW Baixeras J : Immigrant Tortricidae: Holarctic versus Introduced Species in North America. *Insects.* 2020;11(9):594. 10.3390/insects11090594 32899282 PMC7564570

[ref-11] Guan D McCarthy SA Wood J : Identifying and removing haplotypic duplication in primary genome assemblies. *Bioinformatics.* 2020;36(9):2896–2898. 10.1093/bioinformatics/btaa025 31971576 PMC7203741

[ref-12] Harry E : PretextView (Paired REad TEXTure Viewer): A desktop application for viewing pretext contact maps. 2022; (Accessed: 19 October 2022). Reference Source

[ref-13] Howe K Chow W Collins J : Significantly improving the quality of genome assemblies through curation. *GigaScience.* Oxford University Press,2021;10(1):giaa153. 10.1093/gigascience/giaa153 33420778 PMC7794651

[ref-14] Kerpedjiev P Abdennur N Lekschas F : HiGlass: Web-based visual exploration and analysis of genome interaction maps. *Genome Biol.* 2018;19(1):125. 10.1186/s13059-018-1486-1 30143029 PMC6109259

[ref-15] Kimber I : 49.155 BF1086 *Hedya salicella* (Linnaeus, 1758). *UK Moths*, 2023; (Accessed: 20 April 2023). Reference Source

[ref-16] Manni M Berkeley MR Seppey M : BUSCO Update: Novel and Streamlined Workflows along with Broader and Deeper Phylogenetic Coverage for Scoring of Eukaryotic, Prokaryotic, and Viral Genomes. *Mol Biol Evol.* 2021;38(10):4647–4654. 10.1093/molbev/msab199 34320186 PMC8476166

[ref-17] Mulhair PO Holland PWH : Evolution of the insect Hox gene cluster: Comparative analysis across 243 species. *Semin Cell Dev Biol.* [Preprint],2022; S1084-9521(22)00357-3. 10.1016/j.semcdb.2022.11.010 36526530 PMC10914929

[ref-18] Rao SSP Huntley MH Durand NC : A 3D map of the human genome at kilobase resolution reveals principles of chromatin looping. *Cell.* 2014;159(7):1665–80. 10.1016/j.cell.2014.11.021 25497547 PMC5635824

[ref-20] Rhie A McCarthy SA Fedrigo O : Towards complete and error-free genome assemblies of all vertebrate species. *Nature.* 2021;592(7856):737–746. 10.1038/s41586-021-03451-0 33911273 PMC8081667

[ref-19] Rhie A Walenz BP Koren S : Merqury: Reference-free quality, completeness, and phasing assessment for genome assemblies. *Genome Biol.* 2020;21(1):245. 10.1186/s13059-020-02134-9 32928274 PMC7488777

[ref-21] Simão FA Waterhouse RM Ioannidis P : BUSCO: assessing genome assembly and annotation completeness with single-copy orthologs. *Bioinformatics.* 2015;31(19):3210–2. 10.1093/bioinformatics/btv351 26059717

[ref-22] Surana P Muffato M Qi G : sanger-tol/readmapping: sanger-tol/readmapping v1.1.0 - Hebridean Black (1.1.0).Zenodo. 2023a; (Accessed: 17 April 2023). 10.5281/zenodo.7755665

[ref-23] Surana P Muffato M Sadasivan Baby C : sanger-tol/genomenote (v1.0.dev).Zenodo. 2023b; (Accessed: 17 April 2023). 10.5281/zenodo.6785935

[ref-25] Uliano-Silva M Ferreira JGRN Krasheninnikova K : MitoHiFi: a python pipeline for mitochondrial genome assembly from PacBio High Fidelity reads. *bioRxiv.* [Preprint],2022. 10.1101/2022.12.23.521667 PMC10354987 37464285

[ref-26] UniProt Consortium: UniProt: a worldwide hub of protein knowledge. *Nucleic Acids Res.* 2019;47(D1):D506–D515. 10.1093/nar/gky1049 30395287 PMC6323992

[ref-27] Vasimuddin Md Misra S Li H : Efficient Architecture-Aware Acceleration of BWA-MEM for Multicore Systems.In: *2019 IEEE International Parallel and Distributed Processing Symposium (IPDPS).*IEEE,2019;314–324. 10.1109/IPDPS.2019.00041

[ref-28] Wellcome Sanger Institute: The genome sequence of the White-backed Marble, *Hedya salicella* (Linnaeus, 1758). European Nucleotide Archive.[dataset], accession number PRJEB43799,2022.

