# Supplementary material for: The genome sequence of the White-backed Marble, Hedya salicella (Linnaeus, 1758)
Source: Wellcome Open Res. 2023 May 15;8:219. [Version 1] doi: 10.12688/wellcomeopenres.19436.1 (PMC11187532; doi:10.12688/wellcomeopenres.19436.1)
Supplement: Peer review history

# Reviewer report: Reviewer response for version 1

**DOI:** 10.21956/wellcomeopenres.21532.r85362

**Published:** 2024-06-21

**Authors:** Thiago Mafra Batista

**Affiliations:** 1 Universidade Federal do Sul da Bahia, Porto Seguro, Bahia, Brazil

The study describes the sequencing, assembly, and annotation of the genome of *Hedya salicella*, a moth found throughout Europe and introduced in North America.

 The authors employed HiFi long-read and HI-C approaches to assemble a chromosome-level genome, along with sequencing the mitochondrial genome. However, I noticed the absence of BUSCO completeness results for protein-coding genes. Additionally, there was a lack of description on how the transcriptome data (used in genome annotation) were generated.

 The authors did not present analyses of repetitive elements. They mention the number of non-coding RNAs (1,706) but do not specify which types were found or how this was determined in the methods section.

 In the "Genome sequence report" section, the first sentence, "The genome was sequenced from one male *Hedya salicella*…," has already been mentioned at the end of the Background section. I suggest rewriting this sentence.

 The study has significant merit for indexing, but I recommend some minor revisions to further improve the quality of the work.

Are sufficient details of methods and materials provided to allow replication by others?

Partly

Is the rationale for creating the dataset(s) clearly described?

Partly

Are the datasets clearly presented in a useable and accessible format?

Yes

Are the protocols appropriate and is the work technically sound?

Yes

Reviewer Expertise:

I am a bioinformatician with experience in the assembly and annotation of fungal and insect genomes, but I do not have expertise in the taxonomy of this particular group. I have evaluated the aspects within my competency.

I confirm that I have read this submission and believe that I have an appropriate level of expertise to confirm that it is of an acceptable scientific standard, however I have significant reservations, as outlined above.

# Reviewer report: Reviewer response for version 1

**DOI:** 10.21956/wellcomeopenres.21532.r85355

**Published:** 2024-06-19

**Authors:** Leonardo Barbosa Koerich

**Affiliations:** 1 Federal University of Minas Gerais, Belo Horizonte, State of Minas Gerais, Brazil

The manuscript entitled “ The genome sequence of the White-backed Marble, *Hedya salicella* (Linnaeus, 1758)”, by Boyes and colleagues, presents the basic data for the sequencing, assembly, and annotation of the  White-backed Marble moth.

 The manuscript presents the genome assembly, with ~740Mb span, reporting on basic statistics on genome and annotation quality. The genome was sequenced following up-to-date protocols, focusing on long-read data (PacBio) to attain complete chromosomes. Although the authors do not provide a comparison of genome and annotation quality with other insect genomes, BUSCOs numbers suggest that the assembly have a good quality (completeness ~98%). 

 Most of the genome statistics is presented in a Snailplot figure, which I find a poor-choice given it is very confusing, specially to readers not used to genomic data. However, the results are well described and easy to follow.

 The manuscript also reports the annotation of 11,961 protein-coding and 1,706 non-coding genes. However, it is not clear if the annotation is of good quality. For such, I recommend the authors to perform a BUSCO analysis on the annotated genes.

Are sufficient details of methods and materials provided to allow replication by others?

Yes

Is the rationale for creating the dataset(s) clearly described?

Yes

Are the datasets clearly presented in a useable and accessible format?

Yes

Are the protocols appropriate and is the work technically sound?

Yes

Reviewer Expertise:

Genomics of insects vector of diseases

I confirm that I have read this submission and believe that I have an appropriate level of expertise to confirm that it is of an acceptable scientific standard.

# Reviewer report: Reviewer response for version 1

**DOI:** 10.21956/wellcomeopenres.21532.r85357

**Published:** 2024-06-05

**Authors:** Jose Ramon Pardos-Blas

**Affiliations:** 1 Department of Chemistry, Hunter college, Research Foundation of the City University of New York (Ringgold ID: 460801), Belfer Research Building, 413 E 69th St, NY., New York, 10021, USA

The authors present a chromosome-level assembly for the moth species *Hedya salicella*. They used PacBio HiFi long-read sequencing and Illumina 10X Genomic reads sequencing to perform the initial assembly. For chromosomal-level scaffolding, they used Hi-C data. In this report, the authors show the quality data of this genome as well as an annotation of the gene models.

 The sequencing techniques used are appropriate as demonstrated by the high contiguity of the scaffolds. Therefore, the data presented here represents a high-quality genome that has great potential to answer questions related to genome evolution, molecular systematics, population genomics and conservation of Lepidoptera.

 However, I found some points that I would like to clarify:

- Separate “ *H. salicella*, based.”
- The use of “complete” when describing the contiguity of the genome (“This high-quality complete genome assembly of *H. salicella*”) is not accurate. I agree that is a highly contiguous genome but the assembly still has gaps. Please, delete the word “complete”.
- It would be also interesting to report the number of gaps across the final genome assembly.
- It is claimed in the manuscript that the assembly is not fully phased. It would be necessary a clearer explanation of this point in the text.
- I am unclear about the idea behind these sentences: “This high-quality complete genome assembly of *H. salicella*, among a phylogenetically diverse set of insect orders, will yield genomes from closely related species, permitting valuable insights into genomic change over shorter time frames.” Suppose the intention was to point out that this genome can help assemble other genomes of closely related species. In that case, I think it is debatable, considering that closely related species within the same family seem to have some degree of variation in the number of chromosomes (see Table 1 in Traut, Sahara, and Marec, 2008). If the intention was to point out that its comparison with closely related species is of interest for genomic analysis, etc., then, I think it might be necessary to rephrase the sentence. The sentence could be rephrased as: This high-quality genome for the species *H. salicella *constitutes a valuable resource for studying genomic evolution over short periods among closely related species.
- The use of the term sequencing depth instead of coverage could be more appropriate when the authors refer to it in the text. Both terms are interchangeable, but I think in this case it refers to the sense of sequencing depth, and therefore it would be more precise (Sims, et al, 2014 [Ref-2]).
- It is mentioned that transcriptomic information was used for genome annotation, but whether these transcriptomes were sequenced from the same individual or from other individuals is not specified. It is also not specified which tissue was used to sequence the transcriptomes. I believe this section needs further clarification.
- The genome's BUSCO score is reported, but not the BUSCO of the annotation. It would be interesting to know this value for the annotation.
- As described in the article, the genome annotation was performed following the Ensembl gene annotation system. Like most current genome annotations, this method requires the annotation of repetitive elements in the genome. Information on repeats such as microsatellites or transposons is highly relevant since it often constitutes a large amount of genomic material in the species, with direct implications during development or evolution. If possible, I think it would be very informative if this report provides information on the annotation of repetitive elements such as the percentage of total elements found and the most frequent transposon families. Although the suggested addition could be out of the scope of this data note, this information is commonly reported in genome analyses. In the case of this *H. salicella*, it might be of great interest considering the disproportionate size of the Z chromosome, which is five times larger than the average of the other pseudochromosomes.

Are sufficient details of methods and materials provided to allow replication by others?

Partly

Is the rationale for creating the dataset(s) clearly described?

Yes

Are the datasets clearly presented in a useable and accessible format?

Yes

Are the protocols appropriate and is the work technically sound?

Yes

Reviewer Expertise:

Genomic, molluscs, zoology, systematics.

I confirm that I have read this submission and believe that I have an appropriate level of expertise to confirm that it is of an acceptable scientific standard, however I have significant reservations, as outlined above.

## References

[1] : Sex chromosomes and sex determination in Lepidoptera. *Sex Dev* .2007;1(6) : 10.1159/000111765 332-46 10.1159/000111765 18391545

[2] : Sequencing depth and coverage: key considerations in genomic analyses. *Nat Rev Genet* .2014;15(2) : 10.1038/nrg3642 121-32 10.1038/nrg3642 24434847

# Reviewer report: Reviewer response for version 1

**DOI:** 10.21956/wellcomeopenres.21532.r75713

**Published:** 2024-05-11

**Authors:** Xiangyu Hao

**Affiliations:** 1 College of Life Sciences, Northwest A&F University, Yangling, Shaanxi, China

The manuscript presents a comprehensive genome assembly and annotation of Hedya salicella, commonly known as the White-backed Marble. The assembly spans 742.3 megabases, with 25 chromosomal pseudomolecules, including the Z sex chromosome, and a fully assembled mitochondrial genome. This study provides valuable genomic resources for understanding the biology and evolution of this species.

Are sufficient details of methods and materials provided to allow replication by others?

Yes

Is the rationale for creating the dataset(s) clearly described?

Yes

Are the datasets clearly presented in a useable and accessible format?

Yes

Are the protocols appropriate and is the work technically sound?

Yes

Reviewer Expertise:

Zoology, Entomology, Genomics

I confirm that I have read this submission and believe that I have an appropriate level of expertise to confirm that it is of an acceptable scientific standard.
